# Relationship between *CETP* gene polymorphisms with coronary artery disease in Polish population

**DOI:** 10.1007/s11033-018-4342-1

**Published:** 2018-09-03

**Authors:** Joanna Iwanicka, Tomasz Iwanicki, Paweł Niemiec, Anna Balcerzyk, Jolanta Krauze, Sylwia Górczyńska-Kosiorz, Anna Ochalska-Tyka, Władysław Grzeszczak, Iwona Żak

**Affiliations:** 1 Department of Biochemistry and Medical Genetics, School of Health Sciences in Katowice, Medical University of Silesia, Medyków Street 18, 40-752 Katowice, Poland; 2 1st Department of Cardiac Surgery/2nd Department of Cardiology, American Heart of Poland, S. A. Armii Krajowej Street 101, 43-316 Bielsko-Biala, Poland; 3 Department of Internal Medicine, Diabetes and Nephrology, School of Medicine and Division of Dentistry in Zabrze, Medical University of Silesia, 3 Maja Street 13-15, 41-800 Zabrze, Poland; 4 Regional Centre of Blood Donation and Blood Treatment in Raciborz, Sienkiewicza Street 3, 47-400 Raciborz, Poland

**Keywords:** CETP, SNPs, Coronary artery disease, Lipids, Linkage disequilibrium

## Abstract

The cholesteryl ester transfer protein (CETP) gene encodes a hydrophobic glycoprotein that plays a crucial role in the reverse transport of cholesterol. The aim of the present study was to determine whether *CETP* polymorphisms (rs1532624, rs247616 and rs708272) are associated with coronary artery disease (CAD) in a Polish population. Serum lipid levels and single nucleotide polymorphisms of *CETP* genes were determined in 494 subjects: 248 patients with premature CAD and 246 blood donors as controls. Selected polymorphisms were examined using TaqMan PCR analysis. We found that CAD risk was significantly higher for CC homozygotes and C allele carriers of the rs247616 polymorphism than for carriers with the T allele (OR 1.89, 95% CI 1.29–2.76, p = 0.001 and OR 1.51, 95% CI 1.14–1.99, p = 0.003) and likewise for the CC genotype of the rs1532624 polymorphism than for those with the A allele (OR 1.59, 95% CI 1.05–2.40, p = 0.026). Moreover, T allele carriers of the rs708272 polymorphism had significantly higher total cholesterol levels compared to CC homozygotes (p < 0.05) in the healthy controls. We also observed an allelic pattern, C_(rs2477616)_C_(rs708272)_C_(rs1532624),_ which increased susceptibility to CAD by 43% (OR = 1.43, 95% CI 1.10–1.85, p = 0.006). In conclusion, the rs247616 and rs1532624 polymorphisms of CETP may modulate the risk of CAD in Polish population.

## Introduction

Coronary artery disease is currently one of the main causes of morbidity and mortality worldwide. Disorders of the lipid metabolism are crucial risk factors predisposing to the development of cardiovascular diseases [1].

CETP protein plays an important role in regulating cholesterol metabolism [2]. CETP is encoded by the gene *CETP* (16q12-21) [3], which is expressed in many tissues, including the liver, spleen, adipose tissue, small intestine, adrenal glands, kidneys, heart, and skeletal muscles [4–6]. CETP is a hydrophobic glycoprotein involved in cholesterol transport from peripheral tissues to the liver. CETP allows mutual exchange of cholesterol esters and triacylglycerols (TG) between different classes of lipoproteins [7]. Cholesterol esters are transferred from high-density lipoprotein (HDL) to low-density lipoprotein (LDL), triglyceride-rich lipoproteins (TRL) chylomicrons, and very low-density lipoprotein (VLDL), while triacylglycerols are transferred from TRL to LDL and HDL [7]. Numerous studies have shown that high gene activity may result in reduced high-density lipoprotein cholesterol (HDL-C) levels [8–11] and, consequently, the development of atherosclerotic lesions and increased risk of coronary artery disease (CAD) [12, 13]. However, not all studies confirm this dependency [14, 15], and combination therapy with statins and fibrates or CETP inhibitors has not produced the expected results in the reduction of cardiovascular events, despite elevated HDL-C levels [16–18]. According to some studies, there is a relationship between some polymorphic variants of *CETP* and CAD [19–21]. However, other authors suggest a lack of association [22–24].

Because the role of *CETP* polymorphisms in the context of CAD risk remains controversial, we decided to investigate whether three selected *CETP* haplotype-tagging polymorphisms (rs1532624, rs247616 and rs708272) influence the risk of the disease in the Polish population. In addition, we analyzed polymorphic variants searching for haplotype blocks and potential interactions of *CETP* gene alleles with traditional CAD risk factors such as smoking, hypertension, and plasma lipid disorders.

## Materials and methods

### Subjects

We enrolled 494 Polish Caucasians in our case-control study. All participants were inhabitants of the Upper Silesia region. The patients group included 248 individuals with angiographically confirmed premature CAD (75 females and 173 males), aged 44.62 ± 5.95 years. The control group consisted of 246 blood donors (71 females and 175 males) with negative familial history of CAD, aged 43.55 ± 6.33 years. CAD subjects were recruited by the same clinician from the First Department and Clinic of Cardiology at the Upper Silesian Center of Cardiology in Katowice and the First Department of Cardiac Surgery at the Upper Silesian Center of Cardiology in Katowice. Controls were selected from blood donors of the Regional Centers of Blood Donation and Blood Treatment in Katowice and Racibórz.

Inclusion and exclusion criteria, details of the medical interview, diagnosis and evaluation, criteria for CAD, myocardial infarction, and risk factors were as described previously [25]. This study was approved by the Ethics Committee of the Medical University of Silesia in Katowice (Poland) and written informed consent was obtained from all subjects.

### Serum lipid measurement

Plasma total cholesterol (TC), HDL cholesterol, and triacylglycerol levels were obtained by enzymatic colorimetric methods (Analco, Warsaw, Poland). LDL cholesterol levels were calculated using the Friedewald formula [26].

### DNA extraction and genotyping

Genomic DNA was extracted from peripheral leukocytes using the MasterPure genomic DNA purification kit (Epicentre Technologies, Madison, WI, USA). The *CETP* SNPs were analysed, namely: rs1532624 (C > A 16:56971567), rs247616 (C > T 16:56955678) and rs708272 (C > T 16:56962376). The polymorphisms were genotyped using the TaqMan®Pre-designed SNP Genotyping Assay Kit (Thermo Fisher Scientific, Foster City, CA, USA). The 20-µl reaction mix consisted of: 1 µl template DNA (15 ng/µl), 10 µl TaqMan®Genotyping Master Mix (Cat. # 4371355), 1 µl probe (TaqMan® Pre-designed SNP Genotyping Assay), and 8 µl deionized water. The probe was diluted in TE buffer (10 mM Tris–HCl (pH 8.0), 0.1 mM EDTA) (1:1) before the reaction. PCR was performed according to the manufacturer’s specifications. Genotyping was performed using a 7300 Real-Time PCR System (Applied Biosystems). Genotyping was successful in 87–98% of participants. Genotyping accuracy was checked by re-genotyping 15% of the samples, and the reproducibility of results was 100%.

### Statistical analysis

Data were analysed using *Statistica 12.0* (STATSOFT, Tulsa, OK, USA) and *SAS 9.1* (SAS Institute Inc., NC, USA) and SNPator [27]. The Shapiro–Wilk test was used to check the normality of distribution. Comparison of quantitative data was performed by Mann–Whitney U test (non-normal distribution) or the Student’s *t* test (normal distribution). Allele frequencies were deduced from the genotype distributions. Hardy–Weinberg equilibrium testing, comparisons of genotype and allele frequencies between cases and control subjects and potential association of the polymorphisms with CAD clinical phenotypes were calculated using the χ^2^ (Chi square test). Statistical significance was accepted at p < 0.05. Odds ratios (OR with 95% confidence intervals) were computed using univariate and multiple logistic regression analyses after adjustment for age, gender, and traditional CAD risk factors. When the number of individuals in any of the analysed subgroups was zero, risk ratio values (95% CI) were used.

Due to the fact that patients with CAD were treated with statins, the association of genotypes and alleles of the studied polymorphisms with the parameters of lipid metabolism was analyzed only for the control group.

Haploview software was used to determine the haplotype blocks. This software uses the haplotype block definition developed by Gabriel et al. [28] and allows for the determination of haplotype blocks on the basis of population data and information about the locus of a given polymorphism in the gene structure. For assessing linkage disequilibrium (LD) measurements, r^2^ and D′ were used. According to the algorithm used, the r^2^ value should not be lower than 70% (0.7). The CorelDraw Home & Student X7 graphic software was used to present the haplotype blocks.

## Results

### Characteristics of the study groups

Table 1 shows the clinical and biochemical characteristics of CAD patients and blood donors. CAD patients had increased total cholesterol, LDL cholesterol, and triacylglycerol levels, as well as higher body mass index (BMI) values. Furthermore, CAD patients had significantly lower HDL cholesterol levels (Table 1).

**Table 1:** Clinical and biochemical characteristics of CAD patient and blood donor (BD) groups

Characteristics	CAD *n* = 248	BD *n* = 246	OR/RR^a^ (95% CI)	P
Age (years), mean ± SD	44.62 ± 5.95	43.55 ± 6.33	–	NS
Male gender, n (%)	173 (69.76)	175 (71.14)	0.94 (0.64–1.38)	NS
TC (mmol/L), mean ± SD	5.82 ± 1.36	5.09 ± 1.22	–	< 10^−6^
HDL-C (mmol/L),mean ± SD	1.13 ± 0.37	1.46 ± 0.56	–	< 10^−6^
LDL-C (mmol/L), mean ± SD	3.91 ± 1.19	2.98 ± 1.23	–	< 10^−6^
TG (mmol/L), mean ± SD	1.86 ± 0.98	1.38 ± 0.72	–	< 10^−6^
BMI ± SD	27.08 ± 4.26	26.17 ± 3.87	–	0.01
Familial history of CAD, n (%)	174 (72.80)	0	RR = 4.96 (3.98–6.21)	< 10^−6^
Diabetes mellitus, n (%)	22 (8.87)	0	RR = 2.15 (1.95–2.37)	< 10^−6^
Hypertension, n (%)	138 (55.65)	0	RR = 3.51 (2.97–4.15)	< 10^−6^
Cigarette smoking, n (%)	146 (28.87)	67 (27.26)	3.82 (2.62–5.58)	< 10^−7^

*NS* not statistically significant, *OR* odds ratio, *SD* standard deviation, *TC* total cholesterol, *HDL-C* high-density lipoprotein cholesterol, *LDL-C* low-density lipoprotein cholesterol, *TG* triacylglycerol, *BMI* body mass index

^a^Risk ratio values (95% CI), univariate analysis

### Analysis of the CETP polymorphisms

Table 2 shows the genotypes and allele frequencies of the *CETP* polymorphisms. All genotype frequencies conformed to Hardy–Weinberg equilibrium. The frequency of the CC genotype of the rs1532624 polymorphism was higher in the CAD group than in the control group (OR = 1.59, 95% CI: 1.05-2.40, p = 0.026). The frequencies of the CC genotype and C allele of the rs247616 polymorphism were higher in the CAD group than in the blood donor group (OR 1.89, 95% CI 1.29–2.76, p = 0.001 and OR 1.51, 95% CI 1.14–1.99, p = 0.003, respectively).

**Table 2:** Genotype and allele frequencies of *CETP* in CAD patient and BD groups

Genotype/allele	CAD	BD	Inheritance model	OR (95% CI)		P
*CETP rs1532624*						
CC	77 (32.49%)	52 (23.21%)	Dominant versus AC + AA	1.59 (1.05–2.40)	0.026*	
CA	119 (50.21%)	128 (57.14%)	Additive, versus CC	0.63 (0.41–0.97)	0.033*	
AA	41 (17.30%)	44 (19.64%)	Additive, versus CC	0.63 (0.36–1.10)	0.10	
CC + CA	196 (82.70%)	180 (81.08%)	Recessive,versus AA	1.17 (0.73–1.87)	0.52	
C	273 (57.59%)	232(51.79%)	–	1.27 (0.97–1.64)	0.076	
A	201 (42.41%)	216 (48.21%)	–	0.79 (0.61–1.03)	0.076	
*CETP rs247616*						
CC	119 (50.64%)	75 (35.21%)	Dominant, versus CT + TT	1.89 (1.29–2.76)	0.001*	
CT	94 (40.00%)	112 (52.58%)	Additive, versus CC	0.53 (0.36–0.79)	0.002*	
TT	22 (9.36%)	26 (12.21%)	Additive, versus CC	0.53 (0.28–1.01)	0.05	
CC + CT	213 (90.64%)	187 (87.79%)	Recessive, versus TT	1.35 (0.74–2.46)	0.33	
C	332 (70.64%)	262 (61.50%)	–	1.51 (1.14–1.99)	0.003*	
T	138 (29.32%)	164 (38.50%)	–	0.66 (0.50–0.88)	0.003*	
*CETP rs708272*						
CC	80 (33.47%)	63 (26.25%)	Dominant; versus TC + TT	1.41 (0.95–2.09)	0.084	
CT	123 (51.46%)	131 (45.58%)	Additive versus CC	0.74 (0.49–1.12)	0.15	
TT	36 (15.06%)	46 (19.17%)	Additive versus CC	0.61 (0.26–1.07)	0.08	
CC + CT	203 (84.94%)	194(80.83%)	Recessive versus TT	1.49 (0.92–2.41)	0.10	
C	283 (59.21%)	257 (53.54%)	–	1.26 (0.97–1.63)	0.077	
T	195 (40.79%)	223 (46.46%)	–	0.79 (0.61–1.03)	0.077	

*CAD* coronary artery disease patient group, *BD* blood donor group

*Differences statistically significant (p < 0.05)

In the case of the rs708272 polymorphism, there were no statistically significant differences in allele and genotype frequencies between the studied groups. However, we observed an increasing trend in the frequency of CC genotype and C allele in patients compared to that in the controls. These differences were on the border of statistical significance (p = 0.084 and p = 0.077, respectively).

### Association of the CETP polymorphisms with CAD clinical phenotypes

There were no statistically significant associations between *CETP* genotypic variants and myocardial infarction, severe atherosclerosis (presence of multivessel coronary disease or critical occlusion > 90%) observed during coronary angiography, left ventricular hypertrophy, and diabetes mellitus (data not shown).

### Association between genotypes of the CETP polymorphisms and lipid serum concentrations

We analysed the potential interactions between respective *CETP* genotypes and traditional CAD risk factors (male gender, cigarette smoking, hypertension, overweight/obesity, and plasma lipid abnormalities). We did not find any significant differences of lipid levels between respective genotypes of two analysed SNPs; however, we observed that the T allele carriers (TT + CT genotypes) of the rs708272 polymorphism had higher TC concentrations than the CC homozygotes, p = 0.002 (Table 3).

**Table 3:** Lipid profile distribution across genotypes of the *CETP* polymorphisms in BD

BD				
Genotype	TC (mmol/L) ± SD	LD-C (mmol/L) ± SD	HDL-C (mmol/L) ± SD	TG (mmol/L) ± SD
*CETP* rs1532624				
CC	4.78 ± 1.01	2.92 ± 0.95	1.40 ± 0.55	1.40 ± 0.73
CA	5.15 ± 1.25	3.20 ± 1.27	1.46 ± 0.57	1.28 ± 0.66
AA	5.26 ± 1.39	3.24 ± 1.20	1.37 ± 0.52	1.63 ± 0.83
*CETP* rs247616				
CC	4.92 ± 0.99	3.05 ± 0.95	1.40 ± 0.57	1.39 ± 0.77
CT	5.17 ± 1.28	3.16 ± 1.30	1.50 ± 0.57	1.30 ± 0.62
TT	5.37 ± 1.67	3.33 ± 1.44	1.43 ± 0.56	1.63 ± 0.97
*CETP* rs708272				
CC	4.71 ± 0.96^a^	2.90 ± 0.91	1.37 ± 0.47	1.42 ± 0.69
CT	5.15 ± 1.19	3.16 ± 1.20	1.50 ± 0.59	1.33 ± 0.72
TT	5.45 ± 1.47	3.28 ± 1.34	1.46 ± 0.59	1.49 ± 0.76

^a^TT + CT versus CC (p = 0.002)

### Haplotype analysis of the CETP polymorphisms

**Fig. 1:**
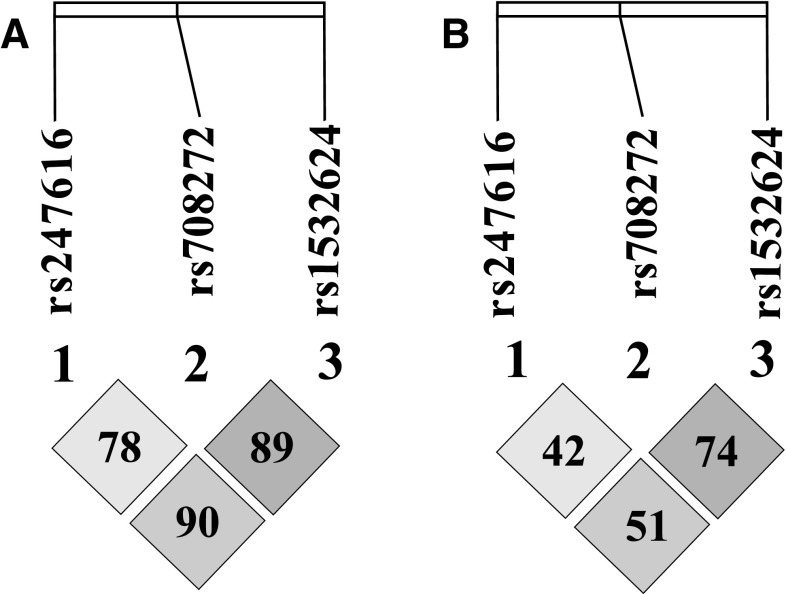
Linkage disequilibrium plot of the *CETP* polymorphisms (**A**—D′ values, **B**—r^2^ values)

Haplotype analysis according to the methods of Gabriel et al. did not show the existence of haplotype blocks between the *CETP* polymorphisms (Fig. 1). Despite the lack of haplotype blocks between the studied polymorphisms, we observed that co-occurrence of alleles C_(rs2477616)_, C_(rs708272)_ and C_(rs1532624)_ increased the risk of CAD by 43% (OR 1.43, 95% CI 1.10–1.85, p = 0.006). Frequency of this allelic pattern was 56% in the CAD group and 47.3% in the blood donors group. The presented results were obtained using the algorithm included in SNPator. We did not observe the impact of co-occurance of this three alleles on plasma lipid values among the healthy controls.

## Discussion

In this study, we investigated three polymorphisms of *CETP* (rs1532624, rs247616 and rs708272) because of their functional significance (the effect of particular alleles and/or genotypes on gene activity) and because their role in the context of CAD is not fully clear.

Analysis of the rs1532624 polymorphism of *CETP* showed a relationship between the CC homozygosity and increased risk of CAD in the studied Polish population sample. The observed result may be caused by the influence of this genetic variant on gene activity and thus the CETP protein concentration. Previous studies showed that people with the AA genotype had lower CETP activity and higher HDL lipoprotein level compared to the carriers of other genetic variants [29]. It can be assumed that the increased gene activity in CC homozygotes results in an increased exchange of triacylglycerols and cholesterol esters between HDL lipoproteins and apolipoprotein B-containing particles. LDL lipoproteins enriched in triacyclglycerols have a longer half-life and longer availability for hepatic lipase [30], which hydrolyzing TG promotes the conversion of LDL to highly atherogenic small dense low-density lipoproteins (sd-LDL) [31]. In addition, the sd-LDL fraction is characterized by increased oxidative susceptibility and greater ability to bind to proteoglycans of the vascular wall [32, 33], which may intensify the atherosclerotic process and contribute to the increased risk of CAD. Since sd-LDL particles are smaller and contain less cholesterol, LDL levels may not reflect increased levels of sd-LDL [34]. This phenomenon may partially explain why we did not observe the association of the studied genotype with LDL level.

In addition, we did not observe a relationship between genotype and HDL concentration, although a previous study by Igl et al. [35] demonstrated the influence of the CC genotype on reduced plasma HDL levels. These differences may be due to different ethnic backgrounds of the respective patient samples, and differences in the inclusion and exclusion criteria of the specific research models. The Igl et al. cohort study took into account statins and factors such as physical activity during work and leisure, as well as diet characteristic for the lifestyle of a particular geographical area. Of course, the proposed pathomechanism and the role of the CC genotype of the rs1532624 polymorphism are purely speculative, and would require experimental confirmation.

In the case of the rs247616 polymorphism, we also showed that CC homozygosity increased the risk of CAD. Previous studies have indicated that the T allele is associated with a decrease in CETP activity and an increase in high-density lipoprotein in Caucasian and English/British populations [36, 37]. Although this polymorphism also did not affect lipid parameters in the current study, we expect that CC homozygosity results in increased CETP activity and consequently increased CAD risk.

Analysis of the rs708272 polymorphism did not show a statistically significant association with CAD but we observed a tendency towards a higher prevalence of C allele in patients than in controls. Our results for the rs708272 polymorphism seem to be partially consistent with the results of some studies. Previous association studies have indicated that the CC genotype is associated with increased risk of CAD [20, 21]. In addition, Ordovas et al. found a protective role for the T allele in relation to CAD as well as a decrease in CETP activity in a subgroup of American males [38]. In context of these studies, association between allele T and elevated TC concentration observed in our analysis is surprising. However, it must be noted that we analyzed an effect of genotype on lipid parameters only in the group of blood donors who had no CAD symptoms and had never been treated with statins and other lipid-lowering drugs.

We note that the results obtained here should be interpreted with caution owing to the limitations of the study, resulting from a relatively small study group, which may weaken the statistical power of potential relationships between the SNPs and the plasma lipid parameters.

## Conclusions

The results of the current study show that the CC genotype of the rs1532624 polymorphism as well as the C allele of and CC homozygosity for the rs247616 polymorphism of *CETP* may be potential risk factors for CAD in the Polish population.
